# Longitudinal dynamics of the nasopharyngal microbiome in response to SARS-CoV-2 Omicron variant and HIV infection in Kenyan women and their infants

**DOI:** 10.21203/rs.3.rs-4257641/v1

**Published:** 2024-04-17

**Authors:** Ayla Žuštra, Victoria R. Leonard, LaRinda A. Holland, James C. Hu, Tianchen Mu, Steven C. Holland, Lily I. Wu, Emily R. Begnel, Ednah Ojee, Bhavna H. Chohan, Barbra A. Richardson, John Kinuthia, Dalton Wamalwa, Jennifer Slyker, Dara A. Lehman, Soren Gantt, Efrem S. Lim

**Affiliations:** Arizona State University; Arizona State University; Arizona State University; Arizona State University; Arizona State University; Arizona State University; Arizona State University; University of Washington; University of Nairobi; University of Washington; University of Washington; University of Washington; University of Washington; University of Washington; Fred Hutchinson Cancer Research Center; Université de Montréal; Arizona State University

**Keywords:** Nasopharyngeal microbiome, SARS-CoV-2, COVID-19, human immunodeficiency virus, Mother-Infant, Genomic epidemiology, Longitudinal

## Abstract

The nasopharynx and its microbiota are implicated in respiratory health and disease. The interplay between viral infection and the nasopharyngeal microbiome is an area of increased interest and of clinical relevance. The impact of SARS-CoV-2, the etiological agent of the Coronavirus Disease 2019 (COVID-19) pandemic, on the nasopharyngeal microbiome, particularly among individuals living with HIV, is not fully characterized. Here we describe the nasopharyngeal microbiome before, during and after SARS-CoV-2 infection in a longitudinal cohort of Kenyan women (21 living with HIV and 14 HIV-uninfected) and their infants (18 HIV-exposed, uninfected and 18 HIV-unexposed, uninfected), followed between September 2021 through March 2022. We show using genomic epidemiology that mother and infant dyads were infected with the same strain of the SARS-CoV-2 Omicron variant that spread rapidly across Kenya. Additionally, we used metagenomic sequencing to characterize the nasopharyngeal microbiome of 20 women and infants infected with SARS-CoV-2, 6 infants negative for SARS-CoV-2 but experiencing respiratory symptoms, and 34 timepoint matched SARS-CoV-2 negative mothers and infants. Since individuals were sampled longitudinally before and after SARS-CoV-2 infection, we could characterize the short- and long-term impact of SARS-CoV-2 infection on the nasopharyngeal microbiome. We found that mothers and infants had significantly different microbiome composition and bacterial load (p-values <.0001). However, in both mothers and infants, the nasopharyngeal microbiome did not differ before and after SARS-CoV-2 infection, regardless of HIV-exposure status. Our results indicate that the nasopharyngeal microbiome is resilient to SARS-CoV-2 infection and was not significantly modified by HIV.

## INTRODUCTION

The nasopharyngeal microbiome plays an important role in overall respiratory health and mucosal homeostasis (1). Interactions and perturbations in the microbiome have been associated with increased susceptibility to respiratory infections, such as influenza (2). The mature adult nasopharynx is colonized by keystone commensal microorganisms such as *Dolosigranulum spp. Moraxella spp*. and *Corynebacterium* spp., as well as traditionally pathogenic microorganisms, namely *Staphylococcus* spp. *Haemophilus* spp., and *Streptococcus* spp. (3–5). The nasopharyngeal microbiome in healthy infants is different than the mature adult microbiome, and ultimately less well-characterized, with conflicting reports on what age the infant nasopharyngeal microbiome begins to resemble the adult microbiome (3). The infant nasopharyngeal microbiome is highly dynamic within the first year of life (4), and is influenced by both host and environmental factors such as antibiotics, birth mode, feeding type, seasonality, and vaccination (5).

Dysbiosis in both the infant and adult nasopharyngeal microbiomes can result in increased susceptibility to severe lower respiratory tract infections (6). Colonization of the early infant nasopharyngeal microbiome by *Streptococcus pneumoniae, Haemophilus influenzae*, and *Moraxella catarrhalis* is shown to increase risk of pneumonia and bronchiolitis, independent of childhood asthma diagnosis (7). Conversely, Respiratory Syncytial Virus (RSV) or Human Rhinovirus (HRV) infection in infants may lead to perturbations in the nasopharyngeal microbiome, which may exacerbate the development of childhood asthma (8). Similarly, in both infants and adults, it has been shown that certain microbiome community states in the nasopharynx are associated with influenza infection susceptibility, though the intricacies of this interaction have yet to be elucidated (2). Overall, there is complex interplay between viral infection, nasopharyngeal microbial composition, and subsequent susceptibility to respiratory infections (6).

Severe acute respiratory syndrome coronavirus 2 (SARS-CoV-2) is the etiological agent of the Coronavirus Disease 2019 (COVID-19) pandemic, and since its emergence in 2019 has evolved multiple variants of concern (VOCs). Delta and Omicron were designated VOCs in May and November 2021, respectively (9).The emergence of Omicron occurred both quickly and abundantly (10, 11), and was first observed in Kenya in November 2021 where it replaced Delta as the primary VOC in the country by mid-December (12). There are functional differences when comparing Delta and Omicron infections. Compared to infections with Omicron variants, those infected with Delta had more severe symptoms for a longer period of time, as well as an increased risk of hospitalization (13). Individuals who were infected with Delta were also more likely to experience long COVID (14). For example, prior immune imprinting has shown those who were infected with a variant other than Omicron, and then reinfected with Omicron had a lessened immune response than those who were initially infected with Omicron and then reinfected with Omicron (15). Furthermore, when infected, individuals were told to self-quarantine at home, which resulted in increased transmission within the home (16). Thus, different VOCs may have distinct impacts on the microbiome.

SARS-CoV-2 infection impact the microbiome and cause dysbiosis. Studies have shown SARS-CoV-2 infection can cause an overall disruption of the gut microbiota marked by a decrease in commensal bacteria and an increase in pathogenic bacteria (17). These disruptions in the gut microbiome can lead to subsequent infections or deterioration of the gut lining, exacerbating the risk for secondary infections (18). The relationship between SARS-CoV-2 infection and the nasopharyngeal microbiome is less clear. Some studies have reported a decrease in specific bacterial species or a decrease in overall alpha diversity (19, 20), while others have reported no significant differences in the nasopharyngeal microbiota (21, 22). Interestingly, studies in which patients had severe COVID-19 symptoms or were hospitalized reported observable differences in specific nasopharyngeal bacterial taxa compared to mild/non-symptomatic cases (23–25). However, studies documenting differences in the nasopharyngeal microbiome were not longitudinal (19, 20), therefore posing challenges in elucidating whether the observed dysbiosis in the microbiome were present pre-infection or a result of COVID-19 infection.

HIV infection and the use of antiretroviral therapies have also been implicated in dysbiosis of the microbiome, with a decrease in bacterial richness and diversity in the gut (26). However, it is not known whether HIV infection or infant exposure to maternal HIV infection influences the nasopharyngeal microbiome, including in the context of SARS-CoV-2 infection. In this study, we aim to characterize the maternal and infant nasopharyngeal microbiome in women living with HIV, HIV-uninfected women, and their infants to determine if SARS-CoV-2 and/or HIV has an impact on the nasopharyngeal microbiome during the peak of SARS-CoV-2 Omicron transmission in Kenya.

## RESULTS

### Study design and SARS-CoV-2 infection in a Kenyan mother-infant cohort from September 2021 – March 2022

**Figure 1:**
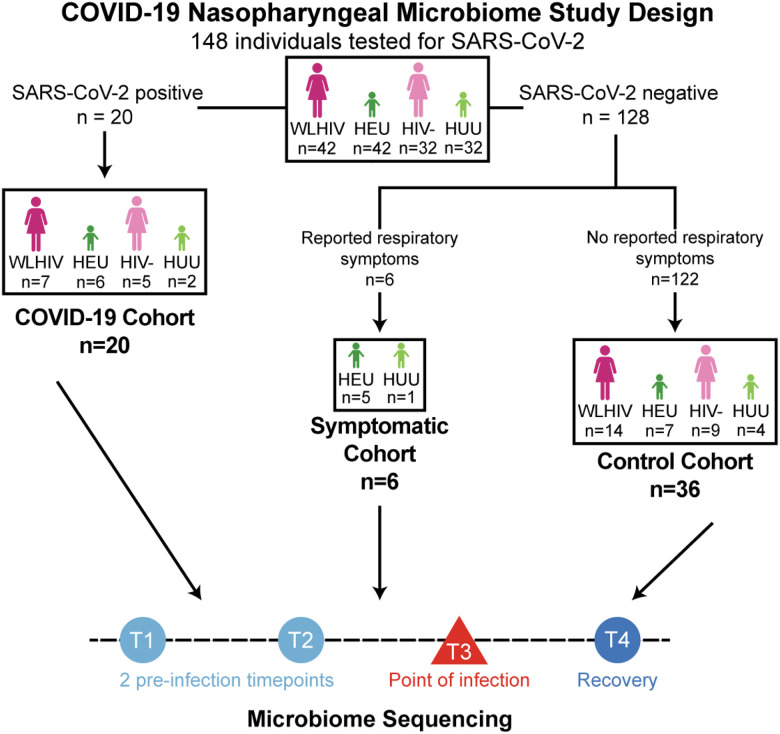
COVID-19 Nasopharyngeal Microbiome Study Design. Overview of study design.

We performed a nested study of Kenyan women and their infants enrolled in a prospective cohort in Nairobi, Kenya who agreed to SARS-CoV-2 testing. Seventy-four mother-infant pairs were included here: 42 women living with HIV (WLHIV), 32 HIV-uninfected women, and their 74 infants: 42 HIV-exposed uninfected (HEU) infants, 32 HIV-unexposed uninfected (HUU) infants (Fig. 1 and **Fig. S1**). WLHIV are given cotrimoxazole to prevent opportunistic infections during high-risk periods, such as pregnancy and breastfeeding. Additionally, HEU infants are given cotrimoxazole as a prophylactic from the age of 4–6 weeks old until cessation of breastfeeding and confirmed HIV-uninfected, typically around 18 months, though this age may vary (27).

Nasopharyngeal swabs were collected for COVID-19 testing (via TaqPath 2.0 qRT-PCR assay) from September 2021 to March 2022 resulting in a total of 1,262 unique swabs from 148 individuals available for this study (average of 8.5 swabs per person over 7 months). Among these, 20 individuals (13.5%) had a single swab test positive for SARS-CoV-2 (1.6% of the total samples). The 20 individuals testing positive for SARS-CoV-2 included 7 WLHIV, 5 HIV- uninfected women, 6 HEU and 2 HUU; there were four mother-infant dyads in which both mother and infant were SARS-CoV-2 positive (Fig. 1, **Table S1**). One individual tested positive in October, 3 in late-November, 15 in mid-December, and 1 in January. Six infants negative for SARS-CoV-2 with reported respiratory symptoms at the peak of SARS-CoV-2 cases in Kenya were included as a symptomatic group. Through hybrid capture next-generation-sequencing, we determined of the six symptomatic infants, one infant was infected with WU Polyomavirus, 4 infants were infected with different strains of Human Adenovirus, and two infants we were unable to determine an etiology (**Table S2**). Additionally, 34 individuals that tested negative for SARS-CoV-2 infection at all timepoints were included as a comparator group: 14 WLHIV, 32 HIV-uninfected women, 7 HEU and 4 HUU (**Fig. S2**). This cohort is unique in that the women and infants were prospectively sampled longitudinally during the COVID-19 pandemic, allowing us to document changes in the nasopharyngeal microbiome before, during and after SARS-CoV-2 infection.

**Figure 2:**
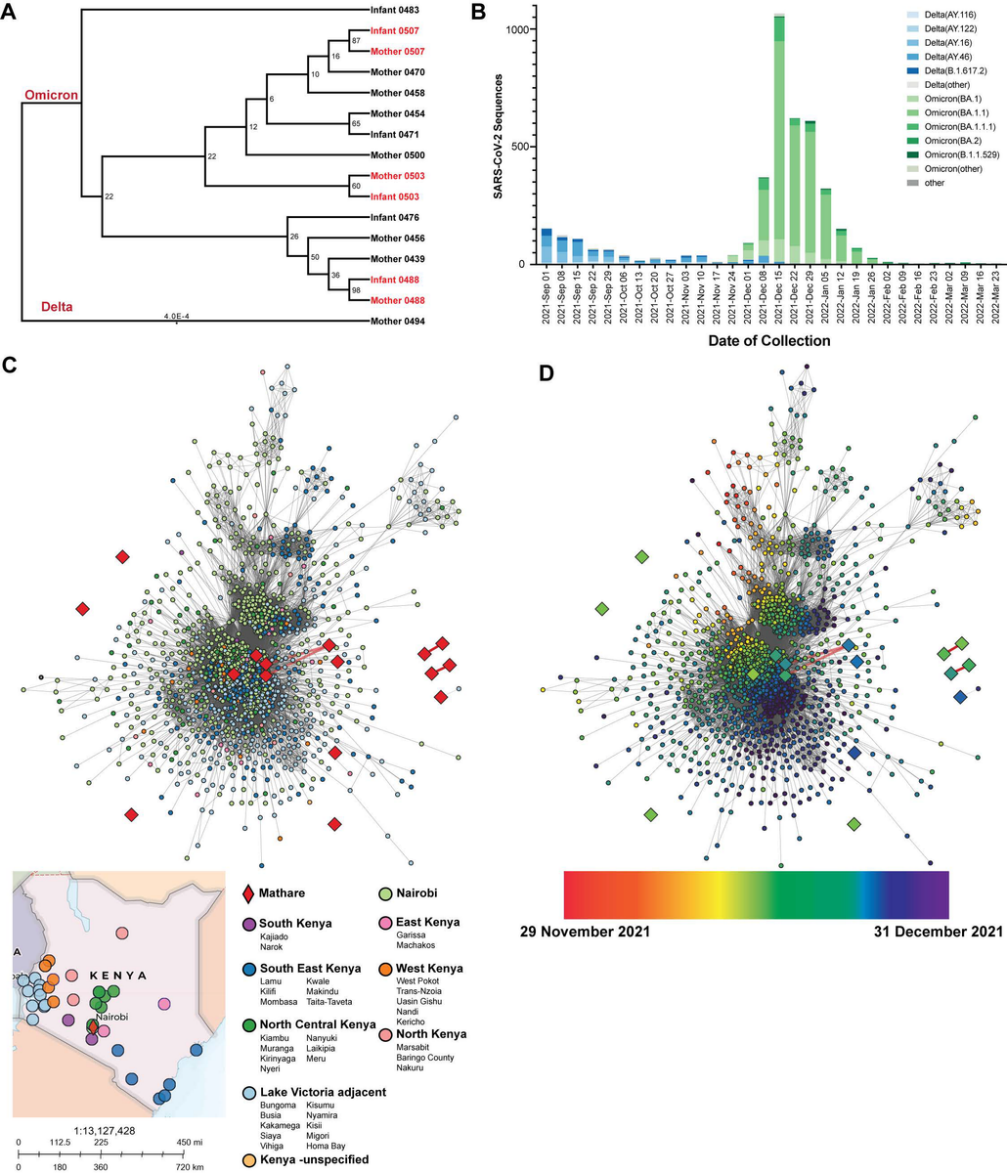
SARS-CoV-2 variant infections among study participants in Mathare and relationship with transmission patterns in Kenya. (A) Maximum likelihood phylogenetic tree of positive SARS-CoV-2 individuals with successful lineage determinations. Mother-infant dyads are highlighted in red. Delta manually designated as outgroup. (B) Sublineage frequencies of circulating SARS-CoV-2 genomes shown by weekly collection date in Kenya. (C) Kenya country map showing geographic areas where sequences were reported. 36 different cities were categorized into the following groups: North Kenya, North Central Kenya, East Kenya, West Kenya, Lake Victoria adjacent, South Kenya, and Southeast Kenya. Nairobi was considered its own group due to sequence abundance. Mathare was considered an independent group. Sequences in which the geographic metadata was not specified beyond Kenya were grouped independently as well. Visualization of all submitted SARS-CoV-2 genomes in Kenya of the generated transmission network. Nodes are colored by geographic area, provided by GISAID reported metadata, and grouped into larger clusters for visualization purposes. (D) Visualization of all submitted SARS-CoV-2 genomes in Kenya of the generated transmission network. Nodes are colored continuously by collection date, provided by GISAID reported metadata, beginning November 29^th^ and ending December 31st 2021.

We first sought to determine which SARS-CoV-2 variants caused infection in the cohort. Through whole genome sequencing, we identified 15 individuals infected with the Omicron variant, one individual infected with the Delta variant, and four individuals for whom there was insufficient genome coverage to successfully determine the variant. We constructed a phylogeny using the 16 SARS-CoV-2 sequences (Fig. 2A). Sequences from three mother-infant dyads included in the phylogeny suggest that the mothers and infants who were SARS-CoV-2 positive at the same time were infected with the same specific strain of SARS-CoV-2.

To compare results of specific SARS-CoV-2 variant transmission in our Nairobi-based cohort to sequences circulating more-widely in Kenya, we performed a sequence network transmission analysis using the sequences from our cohort and 3,016 SARS-CoV-2 sequences from 36 different Kenyan cities and counties submitted to GISAID during the peak of COVID-19 cases both in Kenya and in the study cohort (November 2021 - December 2021; Fig. 2B). November and December 2021 were when most of the study cohort was positive for COVID-19 as well, showing the epidemiology seen in the cohort is comparable to national Kenyan statistics. To examine the introduction of SARS-CoV-2 throughout Kenya, we analyzed the collection dates and locations of samples from GISAID within sub-lineages. There is no clear clustering by geographic area (Fig. 2C), but when collection date information is added, we observe samples from the Nairobi region coinciding with earlier timepoints (Fig. 2D). This would support a scenario where the Nairobi region served as the primary point of SARS-CoV-2 ingress and was spread to the surrounding regions. These data show the cohort samples were consistent with contemporaneous variants circulating in Kenya during the study period.

#### The nasopharyngeal microbiome community states in women and infants

**Figure 3:**
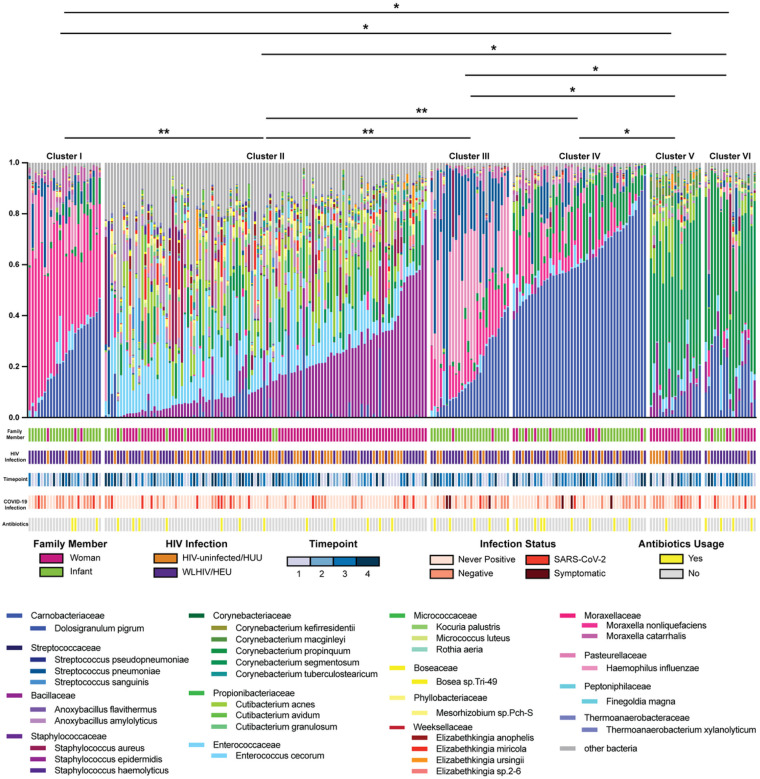
Nasopharyngeal Microbiome Community States. Relative abundance of bacteria at species level, clustered using k-means. Plot labeled with community state clusters. (**) signifies p-values of 0.01 and (*) signifies p-values of 0.05. All statistical differences between clusters are due to differences in woman and infant samples.

To determine the community state profiles present in the mothers and infants, we clustered the nasopharyngeal bacterial microbiome abundance at the species level using the k-means method resulting in 6 clusters (Fig. 3). Cluster 2 was the largest cluster with 106 samples and was dominated by *Staphylococcus epidermidis* (18%) and *Enterococcus cecorum* (16%). Clusters 1, 3, and 4 were dominated by *Dolosigranulum pigrum* (23%, 17%, and 62% respectively). Clusters 1 and 3 were also dominated by *Moraxella nonliquefaciens* (45% and 12% respectively), and cluster 3 additionally was 33% *Haemophilus influenzae* and 24% *Streptococcus pneumoniae*. Cluster 5 was predominantly *Corynebacterium segmentosum* (48%), and cluster 6 was dominantly *Corynebacterium propinquum* (42%). Multinomial logistic regression indicated that all community state clusters were associated with woman/infant status. Specifically, clusters 1, 3, and 4 were majority infant samples (88%, 92%, and 77% respectively), while clusters 2, 5, and 6 were majority woman samples (90%, 94%, and 59% respectively, Fig. 3, see green versus pink in first row below the abundance plots, p-values ranging from 0.01–0.05). None of the community states were associated with HIV-infection (row 2), timepoint (row 3), SARS-CoV-2 infection (row 4), or antibiotics usage (row 5). Taken altogether, these results indicate that community state is most influenced by woman-infant status, and further analyses should be stratified by woman-infant sample.

#### Diversity and richness of the nasopharyngeal bacterial microbiome in women and infants

**Figure 4:**
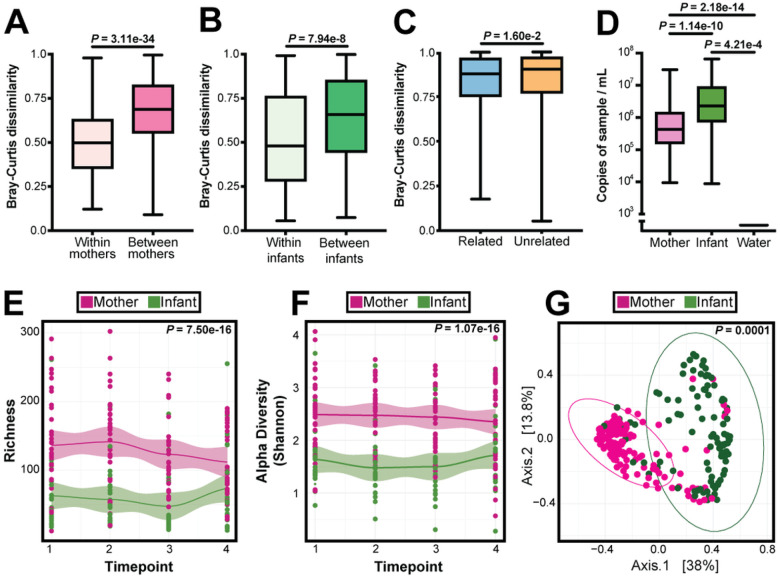
Microbiome Analysis of Mothers and Infants. (A) Bray-Curtis distance for species within individual mothers and between mothers. Statistical significance assessed by Mann-Whitney test. (B) Bray-Curtis distance for species within individual infants and between infants. Statistical significance assessed by Mann-Whitney test. (C) Bray-Curtis distance for species within related mother-infant and between unrelated mother-infant. Statistical significance assessed by Mann-Whitney test. (D) 16S rRNA gene quantitative PCR. 16S rRNA gene copes per sample were quantified in the nasopharyngeal swab samples and water (negative control) samples. Statistical significance was assessed by Mann-Whitney test, corrected for multiple comparisons using Benjamini-Hochberg. (E) Loess plot of bacterial richness against timepoints in mothers and infants. Statistical significance assessed by linear mixed model. (F) Loess plot of alpha diversity against timepoints in mothers and infants. Statistical significance assessed by linear mixed model. (G) PCoA comparing mother and infant samples using weighted Bray-Curtis distance. Statistical significance was assessed by PERMANOVA.

We next considered within-person and between-person microbiome variation between women, infants, and family dyads. Beta diversity was greater when comparing samples between different women than when comparing all samples from an individual woman (p-value = 3.11e-34; Fig. 4A). Similarly, beta diversity was significantly greater when comparing samples between different infants than when comparing all samples from an individual infant (p-value = 7.94e-8; Fig. 4B). In addition, when comparing women and infants using Bray Curtis distance, we observed high dissimilarity between women and infants; however, related women and infants were more like one another than unrelated women and infants (p-value = 1.6e-2; Fig. 4C). Together, these results show that infants and women have lower within-person and within-family variation than between-person variation.

We then considered the differences in bacterial biomass (assayed by 16S qPCR), richness, and alpha diversity between infants and women. Infants had significantly higher bacterial biomass than women (p = 1.39e-10; Fig. 4D). However, women had significantly higher richness (p-value = 7.50e-16) and alpha diversity than infants (p-value = 1.07e-16), which was consistent over time (Fig. 4E and Fig. 4F). Additionally, PCoA analysis of weighted Bray-Curtis dissimilarity showed clearly distinct clustering of women’s samples apart from infant’s samples (PERMANOVA, p-value = 0.001; Fig. 4G). Taken together, these data suggest the infant nasopharyngeal microbiome is more densely populated with fewer bacterial species, whereas the microbiome of adult women is richer and more diverse with less overall biomass, and that these differences were stable over time.

#### Impact of HIV on nasopharyngeal microbiome

**Figure 5:**
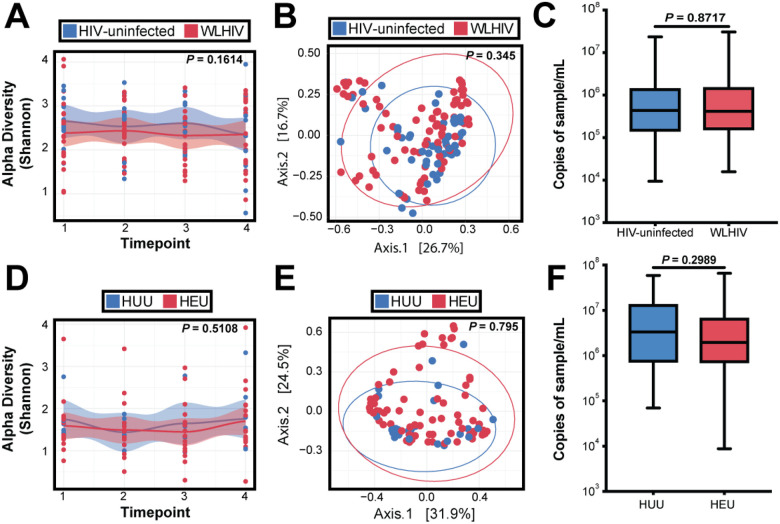
HIV-infection and stability in the nasopharyngeal microbiome. (A) Loess plot of alpha diversity against timepoints in women color-coded by HIV status. Statistical significance assessed by linear mixed model. (B) PCoA comparing WLHIV/HIV-negative women samples using weighted Bray-Curtis distance. Statistical significance was assessed by PERMANOVA. (C) 16S rRNA gene qPCR bacterial load in WLHIV/HIV-negative women. Statistical significance was assessed by Mann-Whitney test. (D) Loess plot of alpha diversity against timepoints in infants color-coded by HIV status. Statistical significance assessed by linear mixed model. (E) PCoA comparing HEU/HUU samples using weighted Bray-Curtis distance. Statistical significance was assessed by PERMANOVA. (F) 16S rRNA gene qPCR bacterial load in HEU/HUU. Statistical significance was assessed by Mann-Whitney test.

We next sought to determine whether HIV infection has an impact on the nasopharyngeal microbiome of women and infants. HIV-uninfected women consistently had higher alpha diversity than WLHIV. However, the difference was small and not statistically significant (p-value = 0.1614; Fig. 5A). PCoA analysis of weighted Bray-Curtis dissimilarity (Fig. 5B), did not show clustering based on HIV status (PERMANOVA, p-value = 0.345) in women. Additionally, there was no difference in bacterial biomass between WLHIV and HIV-uninfected women (p-value = 0.8717; Fig. 5C). Similarly, in infants, we saw no change in alpha diversity (p-value = 0.5108) between HEU and HUU infants (Fig. 5D), and there was no clustering by HIV-exposure status (PERMANOVA, p-value = 0.795; Fig. 5E). There was also no difference in bacterial biomass between HEU and HUU infants (p-value = 0.2989; Fig. 5F). Together, these results suggest living with, or exposure to, maternal HIV infection treated with optimized, long-term antiretroviral therapy does not have an impact on the nasopharyngeal microbiome.

#### Longitudinal dynamics of the nasopharyngeal microbiome and the impact of SARS-CoV-2

We then examined whether temporal changes in the linear trajectories (slopes) of alpha diversity and richness of the nasopharyngeal microbiome were associated with incident SARS-CoV-2 infection, HIV exposure, or antibiotic use. Analyses were conducted separately for women and infants. Among women, neither richness nor alpha diversity changed significantly over time and there was no association between SARS-CoV-2 infection, or antibiotics usage (p-values ranging from 0.0897–0.9277). There was a significant association between richness and HIV status in WLHIV (p = 0.0494), but the relationship was not significant in alpha diversity. In infants, there was also no significant change over time in richness or alpha diversity, and neither SARS-CoV-2 infection nor HIV infection were associated with change in alpha diversity or richness (p-values ranging from 0.1002–0.8819). Convergence of the infant nasopharyngeal microbiome to that of women is expected, but the period in which the infants were sampled was too short to detect the infant microbiome reaching adult maturity. There was an association between alpha diversity and antibiotics usage (p = 0.0289), but this significance was not seen in bacterial richness. Together these data suggest that the infant and maternal nasopharyngeal microbiomes were stable throughout the period of observation and were not influenced by SARS-CoV-2 infection, maternal HIV status or infant HIV exposure or antibiotic use.

While the overall dynamics were not impacted by SARS-CoV-2, we next compared alpha diversity in pre-infection timepoints to the first SARS-CoV-2 positive timepoints to assess transient changes in the nasopharyngeal microbiome. We also compared the nasopharyngeal microbiome before, during, and after SARS-CoV-2 infection between SARS-CoV-2 positive individuals to controls that never acquired SARS-CoV-2 infection. To do so, a longitudinal sampling timeline was constructed using the nasopharyngeal swabs, with two timepoints from the 2 weeks pre-infection, one timepoint during documented SARS-CoV-2 infection and one timepoint an average of 38 days post-infection (**Fig. S2A**). All individuals positive for SARS-CoV-2 had no reported respiratory symptoms at the time of infection. For the controls, the timing was similar, but we substituted the SARS-CoV-2 infection sample with either a timepoint at which the mother or infant had reported respiratory symptoms (**Fig. S2B**), or a matched calendar timepoint if there were no reported respiratory symptoms (**Fig. S2C**).

**Figure 6:**
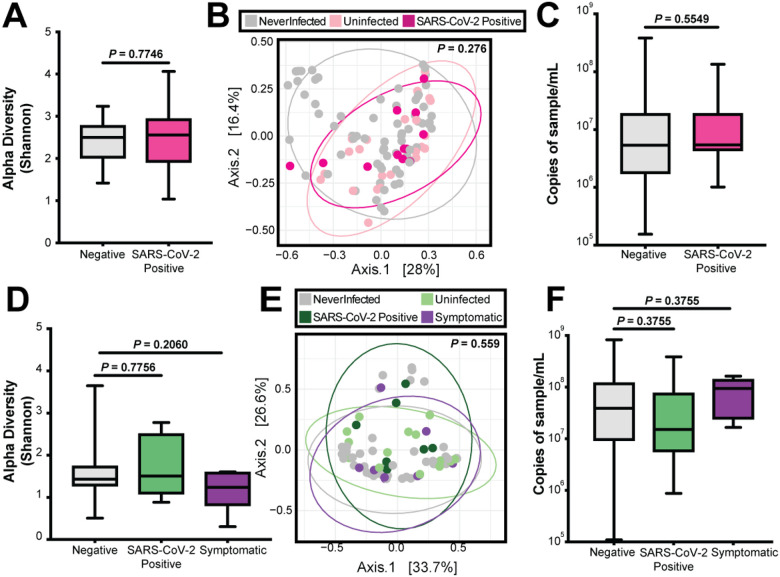
SARS-CoV-2 impact on the nasopharyngeal microbiome. (A) Alpha diversity in women comparing all negative samples to first positive SARS-CoV-2 sample. Statistical significance was assessed by Mann-Whitney test. (B) PCoA in women comparing all never positive, negative, and SARS-CoV-2 positive samples. Statistical significance was assessed by PERMANOVA. (C) Bacterial 16S rRNA gene qPCR bacterial load in women comparing all negative samples and SARS-CoV-2 positive samples. Statistical significance was assessed by Mann-Whitney test. (D) Alpha diversity in infants comparing all negative samples to first positive SARS-CoV-2 sample and first symptomatic sample. Statistical significance was assessed by Mann-Whitney test, correcting for multiple comparisons using Benjamini-Hochberg. (E) PCoA in infants comparing all never positive, negative, SARS-CoV-2 positive, and symptomatic samples. Statistical significance was assessed by PERMANOVA. (F) 16S rRNA gene qPCR bacterial load in infants comparing all negative samples, SARS-CoV-2 positive samples, and symptomatic samples. Statistical significance was assessed by Mann-Whitney test correcting for multiple comparisons using Benjamini-Hochberg.

In women, there was no significant difference in alpha diversity (p-value = 0.7746) when comparing pre-infection timepoints to the timepoint of SARS-CoV-2 infection (Fig. 6A). These results were also supported by PCoA of weighted Bray-Curtis dissimilarity, which did not show clustering of negative samples from never positive women, negative samples from women with SARS-CoV-2 infection, or SARS-CoV-2 positive samples (PERMANOVA, p-value = 0.276, Fig. 6B). There was also no significant difference (p-value = 0.5549) in bacterial load when comparing womens’ negative samples to positive SARS-CoV-2 samples (Fig. 6C). Similarly, there was no statistically significant difference in alpha diversity among infants when comparing negative timepoints to SARS-CoV-2 infected timepoints (p-value = 0.7756). Nor was there a difference between negative timepoints and symptomatic timepoints (p-value = 0.2060) (Fig. 6D). There was no clustering of negative samples from never positive infants, negative samples from infants with SARS-CoV-2 infection, SARS-CoV-2 positive samples, or samples from timepoints with reported respiratory symptoms (PERMANOVA, p-value = 0.559, Fig. 6E). Lastly, we compared bacterial load at pre-infection timepoints, SARS-CoV-2 timepoints, and symptomatic timepoints (Benjamini-Hochberg corrected p-values = 0.3755) in infants (Fig. 6F). Taken together, we conclude that neither SARS-CoV-2 infection nor undiagnosed respiratory infections caused dysbiosis of the nasopharyngeal bacterial microbiome from both negative samples and never positive samples.

**Figure 7:**
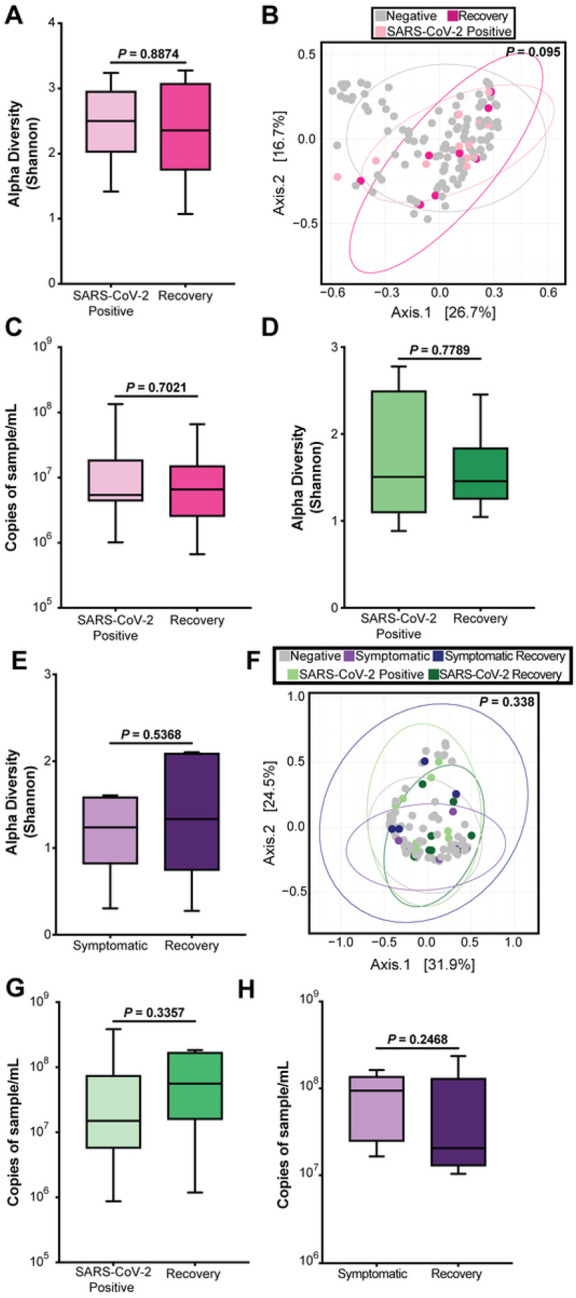
Recovery of the nasopharyngeal microbiome post-infection. (A) Alpha diversity in women comparing first positive SARS-CoV-2 sample to first negative sample post infection. Statistical significance was assessed by Mann-Whitney test. (B) PCoA in women comparing negative, SARS-CoV-2 positive, and recovery samples. Statistical significance was assessed by PERMANOVA. (C) Bacterial 16S rRNA gene qPCR bacterial load in women comparing SARS-CoV-2 positive samples and recovery samples. Statistical significance was assessed by Mann-Whitney test. (D) Alpha diversity in infants comparing first positive SARS-CoV-2 sample to first negative sample post infection. Statistical significance was assessed by Mann-Whitney test. (E) Alpha diversity in infants comparing first symptomatic sample to first non-symptomatic sample. Statistical significance was assessed by Mann-Whitney test. (F) PCoA in infants comparing negative, SARS-CoV-2 positive, symptomatic, and recovery samples. Statistical significance was assessed by PERMANOVA. (G) 16S rRNA gene qPCR bacterial load in infants comparing first positive SARS-CoV-2 sample to recovery. Statistical significance was assessed by Mann-Whitney test. (H) 16S rRNA gene qPCR bacterial load in infants comparing symptomatic sample to recovery. Statistical significance was assessed by Mann-Whitney test.

Though we did not detect a change in the nasopharyngeal microbiome at the time of SARS-CoV-2 infection, SARS-CoV-2 can cause long term sequalae led us to ask whether there were effects on the microbiome after infection. The next available subsequent follow-up sample (average 38 days post-infection) was termed the “recovery” timepoint, and all recovery timepoints were negative for SARS-CoV-2 RNA. The alpha diversity of the nasopharyngeal microbiome remained stable in women even after SARS-CoV-2 recovery (p-value = 0.8874, Fig. 7A). PCoA on weighted Bray-Curtis dissimilarity showed no discernable clustering (PERMANOVA, p = 0.095) in women when comparing negative, SARS-CoV-2 samples and recovery samples (Fig. 7B). There was also no statistical difference (p-value = 0.7021) between SARS-CoV-2 infected timepoints and recovery timepoints when looking at bacterial load (Fig. 7E). Neither infants infected with SARS-CoV-2 (p-value = 0.7789, Fig. 7D) nor infants who were symptomatic for respiratory illness showed a difference in alpha diversity (p-value = 0.5368) from the time of infection to post infection (Fig. 7E). No clustering was apparent with PCoA on weighted Bray-Curtis dissimilarity between negative, SARS-CoV-2 samples, and symptomatic infants, and their respective recoveries (Fig. 7F). Finally, we looked at the differences in bacterial load between infected samples and all available recovery samples. There was no statistical difference in infants between both SARS-CoV-2 infected timepoints and symptomatic timepoints and their respective recoveries (p-values = 0.3357 and 0.2468 respectively, Fig. 7G and Fig. 7H). Taken together, these results show that SARS-CoV-2 infection does not have a lasting impact on the nasopharyngeal microbiome and suggest the nasopharyngeal microbiome is resilient in both mothers and infants, maintaining stability before, during, and after SARS-CoV-2 infection.

#### Specific bacterial species that differentiate the nasopharyngeal microbiome in women and infants

**Figure 8:**
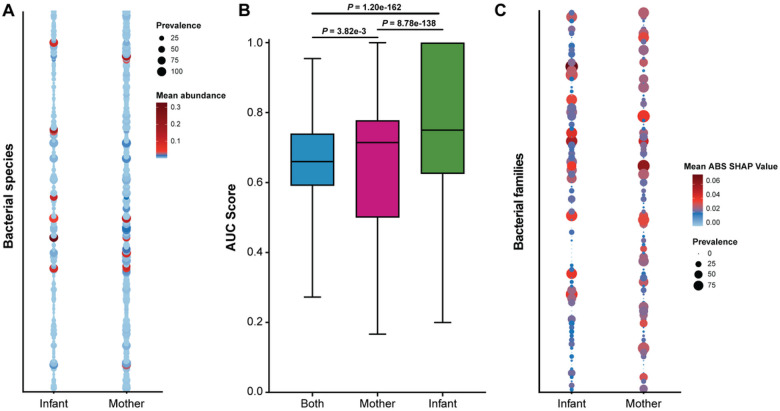
Discriminant analysis on the nasopharyngeal microbiome. (A) Prevalence and abundance plot of statistically significant bacterial species determined by MaAsLin2. Size of circle represents prevalence and color represents bacterial species abundance. (B) Boxplot showing mean AUC scores. (C) Prevalence and abundance plot of statistically significant families determined by ML methods. Size of circle represents prevalence and color represents bacterial family taxa abundance.

Lastly, we performed a multivariate analysis using MaAsLin2 and used novel machine-learning methods to identify discriminating taxa among women and infants and by SARS-CoV-2 infection status. We identified 197 statistically significant discriminating bacterial species (**Table S3**) that differed based on mother-infant status (Fig. 8A). Of these, *Moraxella nonliquefaciens, Moraxella catarrhalis Haemophilus influenzae, Streptococcus pneumoniae, Corynebacterium propinquum* and *Dolosigranulum pigrum* were more common in infants, while *Staphylococcus epidermidis*, *Staphylococcus aureus, Cutibacterum acnes, Corynebacterium segmentosum*, and *Corynebacterium macginleyi* were more common in mothers.

We also used novel machine learning methods to identify discriminating taxa associated with SARS-CoV-2 infection. AUC scores of the best models for combined and stratified datasets confirmed the models should be stratified between women and infants (Fig. 8B). We identified 84 taxa that differed significantly at the familial level according to SARS-CoV-2 infection status (**Table S4**) and plotted the mean Shapley Additive Explanations (SHAP) values for both women and infants (Fig. 8C). Together, these data corroborate our previous finding that the microbiome composition of women is different than that of infants and suggest that SARS-CoV-2 may impact the nasopharyngeal microbiome through smaller compositional changes at the familial taxa level.

## DISCUSSION

In this study, we investigated the impact of SARS-CoV-2 on the nasopharyngeal bacterial microbiome in Kenyan WLHIV, HIV-uninfected women, and their HEU and HUU infants. The nasopharyngeal microbiome was resilient throughout SARS-CoV-2 infection in both mothers and infants, though mothers and infants differed in microbiome composition, diversity, and richness.

We first aimed to elucidate the genomic epidemiology of SARS-CoV-2 within our cohort, and then comparatively to Kenya as a whole. 15 individuals in the cohort were infected with SARS-CoV-2 Omicron, and one individual was infected with SARS-CoV-2 Delta (Fig. 2A).Of note, we found that only 3 mother-infant dyads were positive with the same strain of SARS-CoV-2, indicating the possibility of within-household transmission; the other 14 dyads had discordant strains, demonstrating within household transmission is not inevitable. Proportions of concordant and discordant infections among dyads in this cohort, measured via SARS-CoV-2 serology, has been reported previously, and showed maternal infection was associated with roughly a two-fold increased risk of infant infection (28). Most documented infections in our study cohort were within the same week in December, coinciding with the month Kenya reported the most SARS-CoV-2 cases and deaths (12). By analyzing the transmission networks, these infections were estimated to coincide with the rapid spread of Omicron across the country, rather than geographic location (11, 12) (Fig. 2C and Fig. 2D).

The nasopharynx harbors a bacterial microbiome with core species shared across individuals that are vital to developing a functional respiratory system that is resilient to disease (5), though we observed there are distinct differences in microbial composition between adults and infants (Fig. 3). The infant nasopharyngeal microbiome is predominated by *Dolosigranulum pigrum*, *Moraxella nonliquefaciens*, and *Haemophilus influenzae* (29). *Dolosigranulum pigrum* is a commonly found commensal bacterial in the developing nasopharyngeal microbiome. *Moraxella nonliquefaciens* is common in the developing nasopharynx, though there is conflicting research on whether or not it is a commensal organism or indicative of future dysbiosis or infections (30). While *Haemophilus influenzae* is considered pathogenic, it is commonly colonizes the developing nasopharyngeal microbiome (8). In our study, the female nasopharyngeal microbiome was predominated by *Staphylococcus epidermidis*, *Corynebacterium segmentosum*, and *Corynebacterium propinquum*. Studies have shown species belonging to the *Staphylococcus* and *Corynebacterium* genera are common in the mature nasopharyngeal microbiome (31).

Additionally, we found that the nasopharyngeal microbiome differed between women and infants in richness, diversity, and bacterial load, and that there was less within-person than between-person variation within mothers, infants, and families (Fig. 4A, Fig. 4B, and Fig. 4C). There is no consensus as to when the nasopharyngeal microbiome reaches maturity, the range being anywhere from early childhood to late adolescence (32, 33). Here we are only able to assess the dynamics of nasopharyngeal microbiome maturity during a few months in infants aged 17–25 months. Most studies aiming to characterize the infant microbiome do so with the intent of identifying dysbiosis that may represent further dysbiosis or illness, with much of the data having no clear consensus on what bacteria are pathogenic or commensal (30). As stated previously, the cohort, excluding the six infants who were symptomatic, was largely asymptomatic, including those who were infected with SARS CoV-2. Our study is unique in that we sampled and characterized the stability of the still-developing nasopharyngeal microbiome of healthy infants.

We did not find an impact on the nasopharyngeal microbiome from HIV infection among women receiving antiretroviral treatment or HEU infants (Fig. 5). Studies have reported a decrease in oral microbiota in those who are living with HIV but not receiving antiretroviral therapy (ART); however, the difference was diminished when comparing HIV-uninfected individuals to those receiving ART (34), similar to the comparison groups for our study in which all women living with HIV were receiving long-term, optimized ART regimens. In contrast, studies of WLHIV have reported that antibiotics, but not antiretrovirals have an impact on breastmilk microbiome (35). For those living with HIV today, both HIV infection and immunosuppression are two separate factors considered for overall health. Though we did not have concurrent data to assess the relationship between CD4 count and nasopharyngeal microbiome composition, our data suggest ART-treated HIV infection does not substantially impact the nasopharyngeal microbiome in the presence or absence of SARS-CoV-2 infection. Though, CD4 counts for this particular cohort have been published previously, and were all within the normal range for WLHIV (28).

Multiple studies have looked at the relationship between SARS-CoV-2 infection and the nasopharyngeal microbiome; however, few studies have analyzed this relationship longitudinally. It is important to note that differences in the nasopharyngeal microbiome may vary by geographical location or demographics (25), and no studies to date have focused on Kenyan or African populations. Some studies have suggested that variations in the nasopharyngeal microbiome may be dependent on demographic differences (25). While a few studies (19, 20) found a decrease in alpha and beta diversity in those infected with SARS-CoV-2, others have found the converse to be true (23). Most studies have found no difference in alpha nor beta diversity metrics by SARS-CoV-2 infection status but have noticed dysbiosis in the nasopharyngeal microbiome at the bacterial taxonomic level (36, 37).

Several studies have found an association between SARS-CoV-2 severity and microbiome dysbiosis (24, 25, 38). The cohort we studied reported little to no symptoms throughout the study (28), which is a plausible reason why we observed stability in the nasopharyngeal microbiome. Symptoms are often correlated with increased inflammation and cytokine storms that can potentially cause further dysbiosis to the microbiome (39).

The strengths of our study include the robust longitudinal and cross-sectional sampling of 148 individuals both living with HIV and those HIV-uninfected, as well as individuals infected with SARS-CoV-2 and those who were heathy throughout the duration of the study. To date, there have been no studies focusing on the interplay of SARS-CoV-2 and HIV in the nasopharyngeal microbiome. This is an important area of study given HIV infection may result in more severe SARS-CoV-2 infection (40). Furthermore, being able to study this interaction while being able to compare those who are living with HIV and did not contract SARS-CoV-2, as well as those who are HIV-uninfected and contracted SARS-CoV-2, and those who are HIV-uninfected and never contracted SARS-CoV-2 allows us to investigate all potential viral interactions and their impact on the nasopharyngeal microbiome. The consistent longitudinal sampling also allowed us to truly ascertain that any potential changes in the microbiome were truly a result of SARS-CoV-2 infection and not individual variations in the nasopharyngeal microbiome among individuals. Additionally, our study was able to assess the severity of SARS-CoV-2 infection via reported symptoms at the time of infection. As stated previously, there is a relationship between disease severity, as monitored by symptoms, and nasopharyngeal microbiome disruption (24). Our study is unique in that we were able to study a group of individuals who were non-symptomatic not only at the point of infection, but throughout the study period. Limitations of our study include that we were unable to elucidate the exact window of antibiotics exposure for our study. Depending on whether the weekly visit was recorded as a wellness or COVID-19 visit, individuals were not asked at COVID-19 visits if they were on antibiotics. Additionally, as stated previously, WLHIV and HEU infants are often prescribed cotrimoxazole as a prophylactic during periods of high-risk, resulting in the majority of HEU infants being those who were recorded using antibiotics (27). As stated previously, antibiotics and exposure to them has the potential to impact the nasopharyngeal microbiome, particularly in children (5). Therefore, if we were able to most accurately determine when individuals were taking antibiotics, we would be able to control for any confounding as a result.

The nasopharyngeal microbiome is an often-overlooked and under-researched component of the overall human microbiome, garnering increased study interest over the past three years since the declaration of COVID-19 as a global pandemic. Dysbiosis in any microbiome, including the nasopharyngeal microbiome, may give rise to opportunistic infections, as well as potential inflammatory responses that may further impact the microbiome. Resilience in the nasopharyngeal microbiome upon respiratory infection from a pathogen, such as SARS-CoV-2, implies decreased chance of opportunistic infections, and overall positive implications for human health.

## MATERIALS AND METHODS

### Study population

The Linda Kizazi Cohort was accrued to study how HIV exposure modified establishment of the infant microbiome in breastfed children in the era of universal ART; details of cohort and sampling have been previously published (28). Briefly, pregnant women were enrolled at Mathare North Health Centre in Nairobi, Kenya, and followed with their infant from the third trimester to 24 months postpartum. Detailed information was collected at each study visit to determine clinical symptoms/illnesses, medications, and feeding modality. In this nested sub-study, a total of 74 women and their infants provided longitudinal nasal swabs from September 2021-December 2021, and then had 3 month follow up visits throughout March 2022. All samples collected in this time were tested for SARS-CoV-2 RNA (see below). Samples were selected for microbiome analysis to describe changes during incident SARS-CoV-2 infection: two samples were chosen pre-SARS-CoV-2 infection to establish a microbiome baseline, one sample that tested positive for SARS-CoV-2 RNA, and one sample was chosen post-infection to assess impact on the microbiome over time. Human subjects approvals for all study procedures were obtained from the Kenyatta National Hospital-University of Nairobi Ethics and Research Committee (P472/07/2018), the University of Washington Institutional Review Board (STUDY00004006), and the Arizona State University Institutional Review Board (STUDY00008672).

### Nasal swab collection

Mothers self-collected mid-turbinate nasal swab samples from themselves and their infants. At each visit, participants received sample collection kits containing a viscose-tip swab with plastic handle and 3ml of viral transport medium (VTM; Citoswab Collection and Transport Kit, Citoswab, Nanjing, China). Written and picture instructions for proper sample collection were included with the kit and study clinicians provided verbal guidance by phone for the initial sample and as needed thereafter. Participants were instructed to insert the swab tip into one nostril until they felt slight resistance, rotate the swab in a circle inside the nose for about 15 seconds, and then immediately place the swab into the vial of VTM, breaking off the swab handle and sealing the cap. Participants were asked to inform study staff if any VTM spilled or if they touched the swab tip. Samples were stored temporarily at 2–8°C for up to 24 hours, then separated into 3 aliquots of approximately 1ml volume of VTM before long-term storage at −80°C.

### SARS-CoV-2 testing

We performed real-time reverse transcription polymerase chain reaction (RT-PCR) on the eMAG (TNA) extracts using ThermoFisher TaqPath COVID-19 Fast PCR Combo kit 2.0 assay (Waltham, MA) following the manufacturer’s guidelines. The median SARS-CoV-2 viral load for the three genes tested were 25.4 (IQR 22.4–31.9 *N* gene), 26.7 (IQR 24.1–32.1 *ORF1a* gene), and 26.6 (IQR 23.9–30.9 *ORF1b* gene) respectively.

### SARS-CoV-2 sequencing

SARS-CoV-2 sequencing was performed as previously described (11). NGS library preparation for samples was performed using the COVIDSeq Test (Illumina, San Diego, CA, USA) with ARTICv4 and ARTICv4.1 primers (41). Libraries were sequenced on the Illumina NextSeq2000 instrument using 2 × 109 paired end reads. Adapter sequences were trimmed using trim-galore version 0.6.5 (42), aligned to the Wuhan1 reference genome using the Burrows–Wheeler aligner, BWA-MEM version 0.7.17-r1188 (43), and had their primer sequences trimmed using iVAR version 1.3.1 (44). Consensus sequences were generated using samtools (45) and iVAR software packages. Lineage determinations were performed with pangolin software (46). Sequence quality was validated and annotated using VADR version 1.4 (47).

### Respiratory virus sequencing

Samples negative for SARS-CoV-2 but collected at timepoints with any reported symptoms of respiratory infection of which we have highlighted: fever, cough, and/or shortness of breath were enriched for respiratory virus nucleic acids using the Illumina Respiratory Virus Oligo Panel v2 (San Diego, California). Libraries were sequenced on an Illumina NextSeq2000 instrument using 2 × 109 paired end reads. Sequences were trimmed of adapter sequences and host reads were removed using bbtools (48). Reads were aligned to panel genomes using bowtie2 version 2.2.5 (49). Consensus sequences were generated using samtools (45) and iVAR (44) software packages. Samples with over 1,000 counts and genome-wide coverage were considered positive for infection.

### Microbiome extraction and sequencing

750μl of nasopharyngeal (NP) swab in viral transport medium was transferred into a Qiagen PowerBead Tube and centrifuged at 13,000g for five minutes at 4°. 500μl of supernatant was transferred into a bioMérieux eMAG (Marcy-l’Étoile, France) for total nucleic acid (TNA) extraction. DNA extraction was performed on the NP swab pellet using DNeasy PowerSoil kit (Qiagen). Nucleic acid extracts were stored at −80°C then thawed on ice at time of analysis. Negative controls (PBS) were processed in parallel to the nasal swab samples to assess contamination during extraction, amplification, and sequencing.

### Phylogenetic and network analysis

To generate the phylogenetic tree, genome sequences were first aligned using MAFFT software version 7.520 (50) using default arguments. Phylogenetic analysis was performed with IQTREE 2.2.2.3 (51), using default arguments and ModelFinder algorithm (52) and visualized using FigTree v1.4.4 (53). The sole Delta lineage sequence was designated as the outgroup. Viral transmission networks were generated as previously reported (11). 2,336 GISAID submissions were downloaded 20-June 2022 using text and collection parameters. Text: “Africa/Kenya”. Collection: 2021–29-November to 2021–31-December, and “Complete” genome coverage. Briefly, genome sequences were aligned using MAFFT software version 7.520, using default arguments. A maximum likelihood distance matrix was created using IQTREE2 version 2.2.2.3, with the -m TIM + F + R argument. The distance matrix was converted to an edge list, and source/target pairs exceeding a genetic distance of 6E-05 or having collection dates differing by over 7 days were removed. The network layout was arranged in Cytoscape (V 3.10.0) (54) using a perfuse force directed layout with genetic distance as edge weights.

### Bacterial microbiome analysis

Illumina sequencing reads (2×150) paired end reads were quality filtered using BBtools and cutadapt (55, 56). These reads were then input to krakenuniq (57) to output read counts for assigned bacterial taxa. We then used DBSCAN, a clustering algorithm to obtain the cutoff for optimal number of unique k-mer. For our data anything below 1,800 number of k-mers per taxon was removed. We then used R package decontam as previously described (1.20.0) to remove the default called contaminants (58).

### Linear mixed effects and PERMANOVA models

To account for repeated sampling, we used linear mixed-effects (LME) models (R package nlme version 3.1.149) to compare changes in richness and alpha diversity over time (timepoint) for both women and infants. We used PERMANOVA (vegan, permute, and Adonis in R) to assess changes in beta diversity as measured by weighted Bray-Curtis distances. Family member (women or infant), HIV-status, antibiotic use, ever/never SARS-CoV-2 infection, and timepoint-specific SARS-CoV-2 infection status were variables used in all models to account for exposure or possible confounding. For the models two different methods were used to classify SARS-CoV-2 samples: SARS-CoV-2 infection status at sample collection time and if individuals were ever/never infected with SARS-CoV-2. We first performed LME and Adonis models with family member code to see if there was a significant difference between women and infants and stratified for subsequent analyses. Linear and PCoA plots were plotted using ggplot2 (version 3.4.3), and boxplots were made using GraphPad Prism Version 10.0.2.

### Community state analysis

To obtain the community states present in nasal swab samples of this study we clustered our data with k-means method. We used stats function k-means to cluster the relative abundance at family level into six groups. To determine associations between community states and family member, HIV status, timepoint in study, infection status, and antibiotic use, we used R package mclogit (version 0.8.7.2) to perform multinomial logit models with random effects for patient IDs. Benjamini-Hochberg method was used to correct for multiple comparisons. Relative abundance graph was made using GraphPad Prism Version 10.0.2.

### Differential analysis

To find differentiating bacterial, we created models using R package Microbiome Multivariable Association with Linear Models (MaAsLin2, version 1.14.1) with metadata controlling for longitudinal samples per patient. We used the default q-value threshold of 0.25 for significance, then stratified by between women and infant status. We recalculated the q-value using all results output file and used the updated q-value of 0.05 for significance per factor. Differential abundance plots were plotted using ggplot2 (version 3.4.3).

### Quantitative polymerase chain reaction (qPCR) assay

SYBR green quantitative PCR for the16S rRNA gene was performed using primers 515F (5′-GTGYCAGCMGCCGCGGTAA-3′) and 806R (5′-GGACTACNVGGGTWTCTAAT-3′). The 15μL reaction included 5 μL of extracted DNA and 0.4 μl (100μm) of each primer, 10μl of ThermoFisher Fast Sybr green (Waltham, MA) and 4.2μl of water. The following cycling conditions were used: 95°C for 20 s, then 40 cycles of 95°C for 1 s and 60°C for 20 s followed by a melt curve. Samples were tested in two 96-well plates with twelve water-only negative controls and two positive g-Block controls. g-Block nucleic acids were synthesized by IDT (Coralville, IA) using *Verrucomicrobiae bacterium* 16S V4 gene sequence (n = 292). The positive threshold value (< 40) was determined from gBlock serial dilutions of 10^8^-10^1^ genome copies/μL. All water controls were below the limit of detection. qPCR boxplots and corresponding Mann-Whitney tests were done using GraphPad Prism Version 10.0.2.

### Machine learning

Machine learning analyses were performed on the Sol supercomputer at ASU (59). Normalized reads parsed to the familial taxa were used as the input data and went through natural log transformation with an arbitrary minimal pseudo count added. The dataset was stratified according to woman/infant status and SARS-CoV-2 infection status was used as the predicting label. The first three timepoints, including time of infection, were used in machine learning analyses only from those infected with SARS-CoV-2. For each machine learning model, a 4-fold cross-validation was run on each dataset 1000 times independently (60), and MLP archived the best average AUC. The best model went through SHAP analysis 1000 independently to obtain average SHAP scores for important OTUs. Models selected to participate in the GridSearchCV process are: Random Forest, AdaBoost, Support Vector Machine, MLP from scikit-learn (v1.3.2); XGBoost (v1.7.4), LightGBM (v4.0.0), and CatBoost (v1.2) SHAP (v0.42.1). Differential Abundance plots were plotted using ggplot2 (version 3.4.3), and AUC scores were graphed using GraphPad Prism Version 10.0.2..

## Data Availability

Sequence data have been deposited to the NCBI Sequence Read Archive under accession number PRNJA1078389. SARS-CoV-2 genome sequences have been deposited to the GISAID repository under accession EPI_ISL_18915178 - EPI_ISL_18915193.

## IMPORTANCE

The nasopharyngeal microbiome plays an important role in human health. The degree of impact that SARS-CoV-2 infection has on the nasopharyngeal microbiome varies among studies and may be influenced by diverse SARS-CoV-2 variants and variations in the microbiome between individuals. Our results show that the nasopharyngeal microbiome was not altered substantially by SARS-CoV-2 infection nor by HIV-infection in mothers or HIV-exposure in infants. Our findings highlight the resilience of the nasopharyngeal microbiome after SARS-CoV-2 infection. These findings advance our understanding of the nasopharyngeal microbiome and its interactions with viral infections.
